# Occurrence of shale soils along the Calabar-Itu highway, Southeastern Nigeria and their implication for the subgrade construction

**DOI:** 10.1186/s40064-016-1822-4

**Published:** 2016-02-29

**Authors:** Abidemi Olujide Ilori

**Affiliations:** Department of Civil Engineering, University of Uyo, Uyo, Akwa-Ibom Nigeria

**Keywords:** Highway, Subgrade, Geology, Shale, Stabilization, Organic clay, Silt

## Abstract

This study concerned a stretch of 17 km of a 94-km highway alignment in Southeastern Nigeria that has a high incidence of pavement failure arising from subgrade failure. The subgrade of this section of the roadway is composed of Ekenkpon shale, New Netim marl, and Nkporo shale. Under the Unified Soil Classification System, the shales classify as OH (organic clay) and the marl classifies as MH (inorganic silt). Under the American Association of State and Transportation Officials (AASHTO) M 145 soil classification, all these soils classify as A-7-5 soil. Using the AASHTO M 145 group index, none of these soils was considered suitable as subgrade in its native form. Therefore, cement was investigated as a stabilizing agent. Testing demonstrated that 7, 3 and 12 % by weight were the optimum cement contents to reinforce the Ekenkpon shale, New Netim marl, and Nkporo shale, respectively.

## Background

### Introduction

**Fig. 1:**
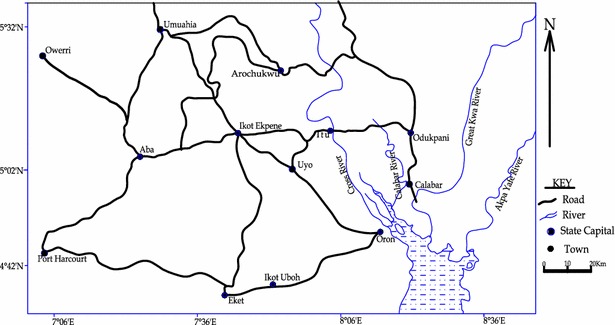
Map of Calabar-Itu highway showing the Calabar-Uyo and Itu-Ikot-Ekpene arms

Shale soil constitutes the dominant soil in Odukpani area of Cross River State, Southeastern Nigeria. Traversing this area is a major two lane highway designated the Calabar-Itu Highway. This highway links two state capitals—Calabar in Cross River State and Uyo in Akwa Ibom State—and continues to the capital city of a third state. A stretch of the highway totaling 17 km (from 24 to 41 km) on the 94-km Calabar-Uyo arm of this highway forms the main focus of study in this article (Fig. 1).

Previous investigations by Akpan and Edet (2005) concerning this highway had indicated that with respect to the underlying geology, frequent pavement failure is characteristic of this section of the highway alignment. The 17-km stretch mentioned above is constructed on predominantly shale and marl soils, while the remaining sections were constructed on Coastal Plain sands and clayey sands of the Benin Formation. This high incidence of pavement failure of this stretch of the highway compared to other parts of the highway necessitated the investigation into the geotechnical properties of the subgrade soil in this section of the highway.

### Study objectives

The objectives of the study were to:

- Identify the major geologic units through which this section of the Calabar-Itu highway alignment traverses that serves as the subgrade for the highway
- Determine geotechnical properties of these geologic units
- Evaluate the suitability or otherwise of the geologic units as subgrade materials.
- Develop a method to improve the subgrade materials strength so as to able to provide long-term support for the pavement structure, if the subgrade soils are found not suitable.
- Develop solutions to avoid or minimize pavement failure arising from the subgrade materials

### Geology and site description of the Calabar-Itu highway alignment

**Fig. 2:**
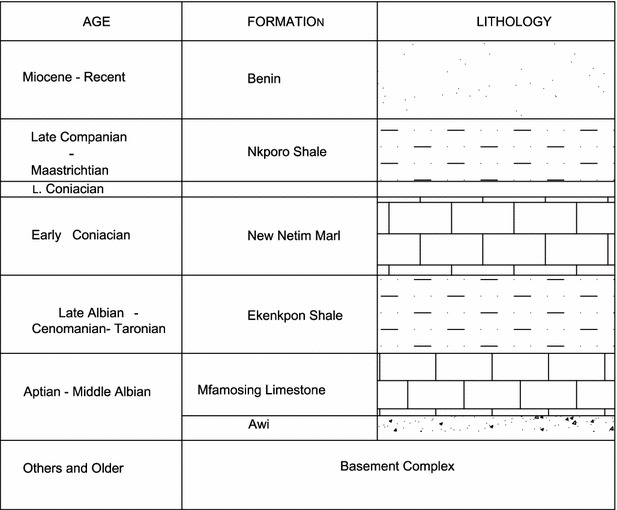
Simplified stratigraphy of Calabar Flank

The general geology of the area that is crossed by the highway alignment has been described by a number of authors. Predominantly, the highway alignment traverses the Calabar Flank, which is described by Ekweme et al. (1995), as that part of the Southern Nigerian Sedimentary Basin which is bounded by the Oban Massif to the north and the Calabar hinge line delineating the Niger delta basin in the south. It is also separated from the Ikpe Platform to the west by a NE–SW trending fault. In the east it extends up to the Cameroon Volcanic ridge. Stratigraphically, the Calabar Flank is composed of predominantly marine sediments approximately 1000 m thick of Albian to Maastrichian (Cretaceous) age. A simplified stratigraphic section of Calabar Flank is shown in Fig. 2.

The earliest sediments deposited within the Calabar Flank are fluvio-deltaic cross-bedded sands of the Awi Formation (Adeleye and Fayose 1978). These are followed by the Mid-Albian Mfamosing Limestone, which represents the first marine incursion into the Calabar Flank (Peters 1982). Carbonate deposition took place on the upland flanks, while shale and calcareous marl were deposited in the intervening depression.

Ekenkpon shale directly overlies the Mfamosing limestone. It is a black, highly fissile shale unit with minor but frequent intercalation of marls, calcareous mudstone, and shell beds. According to Nyong and Ramanathan (1985), the deposition of this shale unit spans late Albian through Cenomanian to Turonian and represents two transgressive cycles.

The Coniacian age New Netim Marl overlies the Ekenkpon shale. The marl is made up of shale fragment, fossil fragments, Calcite and quartz. It is nodular and yellowish. The New Netim Marl is in turn overlain by the Campanian to early Maastrictian age Nkporo Shale. This shale unit is dark grey carbonaceous, friable shale with thin beds of marlstone and gypsum bed (Reyment 1965).

The Miocene to Recent age Benin Formation, which is also called the Coastal Plain Sands, represents the terminal stratigraphic unit in the study area. It comprises mostly of fine to coarse grained, pebbly, moderately sorted sands with local lenses of fine grained, poorly cemented sands and silty clay.

**Fig. 3:**
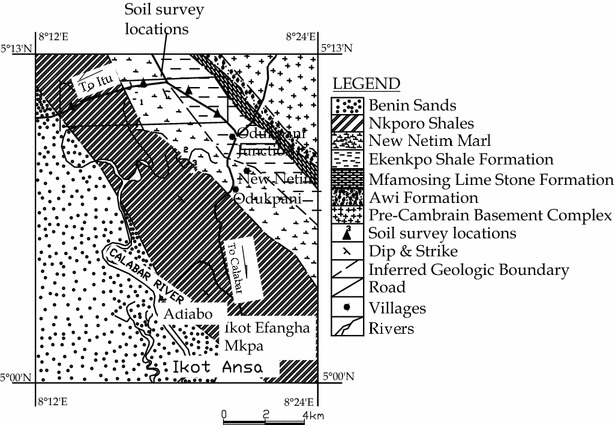
Surface geology of Calabar Flank, Calabar-Itu highway, and soil sample locations

Figure 3 shows the surface geology and the outline of the Calabar-Itu highway through the area. Within the stretch under investigation, the highway traverses the major shale units of the Flank, namely the Ekenkpon shale, Nkporo shale, and New Netim Marls. It is from this area that samples were collected for study.

The Calabar-Itu highway transects two major rivers, the Calabar River, and the Cross River (Fig. 1). The highway is well drained. Rain water does not pond on pavement and shoulders and there is quick runoff of storm water. Ponding of water has not been observed at any location throughout the entire 94-km section of highway. Lack of proper drainage is not considered a cause of pavement failure on any portion of this highway.

## Methods

### Field procedure

Disturbed soil samples were collected from the edge of the pavement at four locations as shown on Fig. 3. The pavement had failed at all these locations.

The fourth location is also Ekenkpon shale, as the highway transects more of this shale unit than others.

### Laboratory tests

Laboratory tests including natural moisture content (NMC), Atterberg limits, sieve analysis, and modified proctor tests, were performed on samples collected from the field. A further classification based on the AASHTO M 145 Standard, which differentiates soils into subgrade and base for the purpose of stabilization was also applied. This classification states that soil with more than 25 % passing a 200-mesh sieve can be regarded as subgrade soil, rather than base soil.

The pH of the soils were determined with a pH meter following the procedure recommended in ASTM D 4972 (2001); however, the soils were soaked in distilled water for up to 72 h rather than 1 h as in the standard. The pH test was carried out to ascertain the relative amount of cement that will be required to stabilize each of the soils economically.

AASHTO M 145-91 (2008) recommends a minimum of 13 % by weight of cement as additive for these types of fine-grained soil. But since the study is to establish a base for these soils, investigations were started with lower cement contents. The plasticity index (PI) and liquid limit (LL) values of the soil are another criterion often used to check whether fine-grained soil is suitable for Portland cement stabilization. Generally, soils with PI values less than or equal to 20 together with LL values not more than 40 are often found suitable for stabilization with Portland cement (NCHRP 2008). None of the soil under investigation met the Liquid Limit criteria, while only Nkporo shale soil failed the plasticity criterion.

California Bearing Ratio (CBR) tests were carried out on soil samples soaked for 24 h. Preliminary CBR values indicated the need to introduce additives to the soil to increase strength. Trial mixes were made with different percentages of cement from 1 to 7 % by weight mixed with the soil samples. These CBR tests were carried out in accordance with the AASHTO T 193-10 (2010) Standards. The Modified AASHTO test, which is the standard adopted for road and highway works locally, was adopted for the study. In preparing the samples for testing, dry ordinary Portland cement was manually but thoroughly mixed with dry soil samples, a measured amount of water that will bring the soil to the Optimum Moisture Content for each of the soil under study the optimum moisture, is added to the soil, while manual mixing of the resulting mixture is carried out. The Ordinary Portland cement additives employed yield a strength of 32.5 N/mm^2^ at 28 days in standard application. To evaluate the effectiveness of a cement-stabilized soil, testing according to the ASTM D 559 (1996), Standard is often used to determine strength and durability of samples subject to wet-dry cycles. However, such tests are user subjective and for that reason were not adopted in this study. Instead, unconfined compressive strength tests were adopted. These tests were carried out on samples treated with various percentages of the cement ranging from 2 to 7 % and containing the optimum moisture content of each soil. The samples were water cured for 28 days at room temperature and soaked for 4 h before unconfined compressive strength testing. The unconfined compressive strength tests are carried out on samples extruded from modified AASHTO compaction mold. The sample specimens from the mold have 14.5 cm diameter and a height of 12.5 cm. Testing was carried out with a motorized loading machine which has a strain rate of 1.27 mm/min, and is equipped with digital deformation and load gauges which were read manually. Readings were taken until the load gauge reading starts to decrease at which point the test ends. Calculations were made that enable a plot of stress versus strain. The peak value at the turn of the resulting curve is the unconfined compressive strength. The maximum Young’s modulus values were estimated using the values of stress at the peak of the plotted curve and zero as the ordinate and the associated strain. The minimum values were estimated from the slope of straight line at the early linear part of the curve. The maximum value of stress used in this computation is taken as the minimum unconfined strength for the sample.

## Results and discussion

### Results of property tests on unmodified soils

#### Soil classification

The results of sieve analysis, natural moisture content, Atterberg limits, and AASHTO M 145 and USCS classifications of the soil samples are presented in Table 1. Nkporo Shale is classified as Organic Clay under USCS classification and A-7-5(20) under the AASHTO soil classification system, with the highest Group index value which represents the poorest subgrade rating. Ekenkpon Shale also classified as OH, while New Netim Marl classified as MH (Inorganic Silt). Values of maximum dry densities (MDD) and optimum moisture contents (OMC) obtained from compaction tests are also presented in Table 1.

**Table 1:** Soil indices, moisture—density values, and classification for native soil

Sample No	Geologic unit	Natural moisture content (NMC) (%)	Plastic limit (%)	Liquid limit (%)	Plasticity index (%)	Liquidity index	Sieve sizes in (mm)		4.75	2.36	1.18	0.6	0.425	0.3	0.15	0.075	pH	AASHTO classification	Unified soil classification system (USCS)
							Maximum dry density (kg/m^3^) (MDD)	Optimum moisture content, (%) (OMC)	Percentage passing sieve no										
										7	14	25	36	52	100	200			
I	Nkporo Shale	26.3	30.0	52.0	22.0	−0.2	1620.0	14.6	100.	93.9	93.9	89.8	88.4	87.3	85.5	80.1	2.27	A-7-5(20)	OH
I	New Netim Marl	15.6	33.0	48.0	15.0	−1.2	2020.0	8.6	94.7	84.0	84.0	79.9	78.0	76.2	72.6	68.4	6.97	A-7-5(11)	MH
I	Ekenkpon Shale	27.8	38.4	52.8	14.4	−0.7	1700.0	16.6	94.0	89.0	89.0	86.6	85.8	84.8	83.0	81.4	4.28	A-7-5(15)	OH
II	Ekenkpon Shale II	26.2	36.5	51.5	15.1	−0.7	1680.0	18.5	100.	99.2	99.2	97.2	95.8	94.6	91.8	90.0		A-7-5(18)	OH

#### California Bearing Ratio

Un-soaked and soaked CBR specimens were tested. Table 2 presents the CBR values for native soil samples at optimum moisture content for both un-soaked and soaked states. These values range from 5 to 26.8 %. None of the soils meet basic requirements [AASHTO, and Government of the Federal Republic of Nigeria (1997) criteria] or soaked CBR value of 30 % for both subgrade and subbase.

**Table 2:** Moisture—density values and California Bearing Ratio for virgin soil

Location	Geologic unit	Optimum moisture content, NMC (%)	Unsoaked CBR at OMC (%)	24 h soaked CBR (%)	Moisture after soaking (%)
A	Nkporo Shale	14.6	35.0	26.8	16.0
B	New Netim Marl	8.6	27.5	20.6	10.0
C	Ekenkpon Shale	16.6	15.0	7.0	22.2
D	Ekenkpon Shale	18.5	14.0	5.0	20.7

### Soil improvement

#### General considerations

Soil modification can increase the strength properties of a subgrade soil and is usually carried out using lime, fly ash or cement, or any combination of the three (Little et al. 2000), These materials are known as traditional stabilizers (NCHRP 2008) because they rely on pozzolanic reaction and cation exchange to modify and/or stabilize soil. Some other materials such as rice husks, sawdust and sawdust ash (SDA) have also been used, mostly in rural, low traffic volume roads. The choice of improvement agent is influenced by the following (NCHRP 2008; Muhunthan and Sariosseiri 2008);

- Soil mineralogy and content (sulfates, organics, etc.…)
- Soil classification (gradation and plasticity)
- Goals of treatment
- Mechanisms of additives
- Desired engineering and material properties (strength, modulus, etc.…)
- Design life
- Environmental conditions (drainage, water table, etc.…)
- Engineering economics (cost vs. benefit)

For the present study, all the above were taken into consideration. Ordinary Portland cement was selected as the improvement agent; lime and fly ash are not readily available in economical quantities whereas the study area has a number of cement factories.

#### Strength and resilient modulus requirements for soil stabilization

AUSTROADS research report AP-R434 (Austroads 2013a) classifies modified or stabilized soil based on water cured 7 and 28-day unconfined compression strength (UCS) values as presented in Table 3. Generally, there are two broad classifications, namely:

**Table 3:** Typical properties of modified, lightly-bound and heavily-bound materials

Material type	Layer thickness (mm)	UCS at 7- days (MPa)	UCS at 28 days (MPa)	Design resilient modulus (MPa)
Modified	Applicable for any thickness	≤1.0	1.3	≤1500
*Bound*				
Lightly -bound	Generally ≤ 250 mm	1–2.0	1.3–3.0	1500–2000
Heavily-bound	Generally > 250 mm	≥2.0	≥3.0	2000–20,000

From AUSTROADS AP R434-13. For slow setting binder the 28 day test results will be less than the values shown but will continue to increase in the field for at least 6–12 months

- Modified material, with a UCS value less than or equal to 1.0 MPa at 7 days or 1.3 MPa at 28 days. The resilient modulus for the soil should be ≤1500 MPa (1.5 GPA).
- Bound material, which is further subdivided into;
  1. Lightly bound, with 7-day UCS between 1 and 2.0 MPa or 28-day UCS between 1.3 and 3.0 MPa with thickness less than 250 mm. The resilient modulus value should range from 1500 to 2000 MPa (1.5–2.0 GPA).
  2. Heavily bound with 7-day UCS value >2.0 MPa or above 3.0 MPa at 28 days with a thickness >250 mm and design deformation modulus between 2000 and 20,000 MPa (2.0–20.0 GPa)

Based on the above and the UCS values listed in Table 4; at optimum cement content (the most economical amount of cement that gives a resulting mix a minimum unconfined compressive strength value of 1.5 MPa), Nkporo shale has a maximum UCS value above 3.4 MPa at 12 % cement content, New Netim Marl 2.17 MPa at 3 % cement content, and Ekenkpon 1.81 MPa at 7 % cement content. Therefore Nkporo shale can be classified as heavily bound, while New Netim Marl and Ekenkpon shale soil can be said to be lightly bound. On the other hand the NCHRP (2008) only distinguished between modified soil and stabilized soil based on UCS value of 1.5 MPa. Based on this criterion all the soils at the optimum cement content are stabilized soils. The AUSTROADS research report, AP-R434 (2013a) classification appears to be superior over the NCHRP (2008), since it further classifies soil into lightly bound and heavily bound based on the UCS, and also recognizes that bound materials contribute significantly to pavement strength by stiffening the pavement foundation. However this stiffening can be accompanied by development of shrinkage cracking. NCHRP (2008) also recognized this and suggested that slow setting additives should be added to such stabilized soil.

**Table 4:** Unconfined compressive strength and Young’s modulus values for the three soils at different cement contents

Location	Geologic unit	Unconfine compression value	0 % Ordinary Portland cement	2 % Ordinary Portland cement	3 % Ordinary Portland cement	5 % Ordinary Portland cement	7 % Ordinary Portland cement	10 % Ordinary Portland cement	12 % Ordinary Portland cement	pH value
A	Nkporo Shale	Lowest q_u_ (kPa)	30.28	30.28	60.56	60.54	121.13	30.27	60.54	2.27
		Peak, q_u_ (kPa)	784.19	604.19	302.22	692.87	531.25	240.99	3414.90	
B	New Netim Marl	Lowest q_u_ (kPa)	48.41	121.08	605.41	666.21	665.14			6.97
		Peak, q_u_,(kPa)	674.53	743.76	2172.48	2498.33	2822.90			
C	Ekenkpon Shale	Lowest q_u_ (kPa)	72.65	30.27	66.62	60.56	181.69			4.28
		Peak (kPa)	350.57	446.73	963.95	845.17	1806.68			
		Young’s modulus values (MPa)								
A	Nkporo Shale	At lowest q_u_	75.71	75.71	151.41	151.41	300.40	37.53	75.68	
		At peak q_u_	178.23	215.78	125.92	115.43	146.39	42.69	304.90	
B	Ekenkpon Shale	At lowest q_u_	40.34	151.35	756.76	1665.53	329.91			
		At peak q_u_	112.42	371.88	543.12	477.50	343.51			
C	New Netim Marl	At lowest q_u_	90.81	37.53	165.22	150.20	450.60			
		At peak q_u_	146.07	123.10	170.76	477.50	298.70			

#### Strength and durability test results

**Fig. 4:**
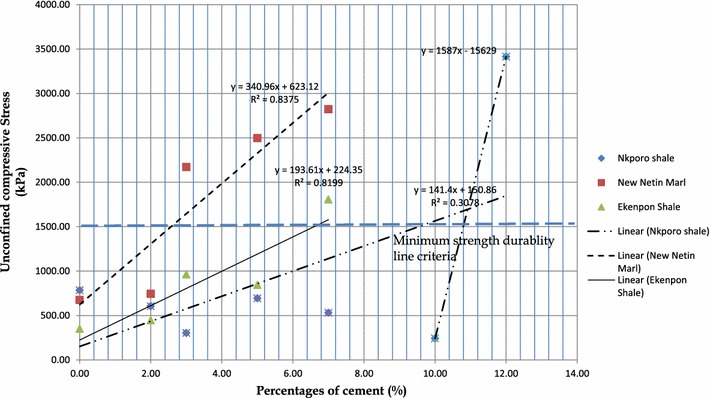
Variation of unconfined compressive strength with cement content for the soils

Table 4 presents the minimum and peak values of unconfined compression tests results for different percentages of cement for each of the soil. Figure 4 shows the variation of unconfined compressive peak strength values for the three soils with various percentages of cement. ASTM D 560 (2003), and Austroads research report AP-R434 (2013a), specify unconfined compressive strength (UCS) value of 1.5 MPa on 28 days moist cured sample. A durability line based on the above requirements is shown in the Fig. 4. The different values of UCS for each soil were plotted against the different percentages of cement. Best-fit straight lines were determined for each soil using least-squares regression. The r^2^ correlation coefficients were 0.84 for New Netim Marl, 0.82 for Ekenkpon shale, and 0.31 for Nkporo shale. The minimum cement content for each soil that will ensure durability of the stabilized soil in the field is found where the correlation line crosses the durability line. These values are, 2.20 % for New Netim Marl and 6.2 % for Ekenkpon shale. The resulting soil mixtures in both cases at the optimum cement content have low plasticity values; tending towards 7 % in case of New Netim Marl and 9 % in case of Ekenkpon shale. Such value could not be put forward for Nkporo shale since the single linear correlation coefficient is poor. Considering the data for Nkporo shale as two sets; the first, from 0 to 7 %, and a second data set for 10 and 12 % cement content. A bilinear correlation for the two sets resulted in a second correlation line that gives optimum cement around 11 %. The maximum value of UCS test for the 10 % is <1.5 MPa, whereas the 3.41 MPa, for 12 % cement content surpassed this minimum value. Also no experiment was carried out for 11 % cement content; therefore 12 % by weight of cement content will serve as the optimum cement content required to stabilize Nkporo shale.

#### Soil indices and stabilization

Table 5 presents variations of Atterberg limits for the three soils with various percentages of cement content. The tests for determination of Atterberg limits were carried out on the 28 day water cured sample. Compared to its virgin sample the Nkporo shale plastic limits fluctuates in values as cement content increases. LL varies with increased cement content from 52 % at zero cement to 37 % at 12 % cement. Plasticity Index (PI) reduces from 22 % at zero cement to 9.01 % at 5 % cement, jumps up to 21.0 % at 10 % cement content, then 11.5 % at 12 % cement content. The increase in plasticity Index at 10 % cement content for the Nkporo shale is likely due to an optimum reaction with sulphate present in the shale (Nganje et al. 2015). Sulphate levels at concentration of 3000 ppm (3000 mg/l) and above (NHRCP 2008) can react with cement to produce a swelling potential that is destructive to pavement. The various degrees of concentration of sulphate measured in ground water (hand dug wells), and surface waters (ponds, streams) within Nkporo shale geologic area reported by Nganje et al. (2015), ranges from 0.00 to 8542.80 mg/l. The value that must be dominant in the Nkporo soil in the area must be less than the threshold value for which the soil reaction with Portland cement will result in destructive expansive potential but significant to raise the PI of the stabilized soil at 10 % cement content, which is reduced when cement reaches 12 %. This trend is similar to the pattern shown by unconfined compressive strength test, the result of which drop to a minimum with progressive increase in cement content that reaches the lowest at 10 % cement content and then increasing significantly at 12 %. An explanation for this behavior trend will be made in the discussions under ‘cement stabilization and pH’.

**Table 5:** Variation of Atterberg limits and plasticity index for the three soils with different percentages of cement content

Geologic unit	0 % Ordinary Portland cement	2 % Ordinary Portland cement	3 % Ordinary Portland cement	5 % Ordinary Portland cement	7 % Ordinary Portland cement	10 % Ordinary Portland cement	12 % Ordinary Portland cement	pH value
Nkporo Shale								
Plastic limit (%)	30.00	–	36.89	28.99	–	18.00	25.50	
Liquid limit (%)	52.00	–	46.00	38.00	–	39.02	37.00	2.27
Plasticity index (%)	22.00	–	9.11	9.01	–	21.00	11.50	
New Netin Marl								
Plastic limit (%)	33.00	33.00	30.00	37.00	30.00			
Liquid limit (%)	48.00	44.00	37.00	46.00	47.00			6.97
Plasticity index (%)	15.00	11.00	7.00	7.00	17.00			
Ekenpon Shale								
Plastic limit (%)	38.40	44.3	40.50	37.40	41.60			
Liquid limit (%)	52.80	73.20	62.20	52.60	40.60			4.28
Plasticity index (%)	14.40	28.90	21.70	15.20	9.00			

The New Netim Marl soil shows progressive decrease in PI from zero cement content up to 5 % cement content by weight of the soil. With values of 15 % at zero cement content to 11 % at 2 % cement content then to 7 % at 3 %,and 5 % of cement content. The value then goes up to 17 % at 7 % cement content indicating a less stiff soil. Analysis of strain existing at the peak stress shows that at 5 % cement content the peak stress is 2498.33 kPa and the associated strain is 8.80 × 10^−03^ and at 7 % cement content the peak stress is 2822.9 kPa and the associated strain is 1.29 × 10^−02^. This strain value is larger than the one at 5 % cement content. The Atterberg limit values for this stabilized soil vary in a fluctuating manner, though in a cyclic pattern, from 48 % at zero cement content to a lowest value of 37 % at 5 % cement content and then up again to 47 % at 7 %.

The Ekenkpon shale soil shows an initial increase in LL at 2 % cement, but further increase in cement content results in continual decrease in LL until 7 % when the liquid limit attained was 40.6 %. The PI follows a similar trend; with a PI of 28 % at 2 % cements content decreasing to 9 % at 7 % cement content.

#### Cement stabilization and pH values

Portland cement is comprised of calcium-silicates and calcium-aluminates that hydrate to form cementious products. Cement hydration is relatively fast and causes immediate strength gain in stabilized layers (Little et al. 2000). The mechanism of Portland cement reaction with soil is well documented. When Ordinary Portland cement is mixed with soil in the presence of adequate moisture, cement hydrates and produces free lime, Ca(OH), which reacts Pozzolanically with soil as long as the environment is alkaline, that is with a high pH value. It therefore follows that a soil with pH value in or towards alkaline region will react more productively in the presence of adequate moisture. Hence the need to determine the pH values of the native soils being investigated.

Soil pH values were; 2.27 for Nkporo shale, 6.7 for New Netim Marl, and 4.28 for Ekenkpon Shale. Typically, a soil with low pH (acidic) will require more cement content for stabilization as its pH will have to be raised to significantly more than ‘7’ before cement will react properly (the pH of cement typically ranges between 9 and 11). Conversely a soil with a pH value towards or in the alkaline region may require less amount of cement than the previous one. The Nkporo shale with a pH of 2.27 did not show any appreciable strength gain with the initial percentages of cement (2, 3, 5, 7 %) used for all the soils. Hence the cement content utilized for the Nkporo shale was increased to 10 and 12 %; whereas New Netim marl with a pH value of 6.7 required only 3 % cement content by weight for stabilization, and Ekenkpon shale with a pH of 4.25 required 7 % by weight of cement. Although the clay mineralogy of a soil also influence the way it will react with cement; the mineral structure of the soils under study were not determined as it was not one of the objectives of this work. Therefore no comment on the influence of clay mineralogy on their stabilization can be made.

#### California Bearing Ratio and cement content

Although the CBR value is not the main criteria used in determining the optimum cement content for stabilization; it was determined for some trial mixes that were soaked for 28 days. Ekenkpon Shale which occurs more extensively in the study area and with a pH value in between the other two was mixed with the trial percentages of cement. Table 6 presents CBR results for these trial mixes. Figure 4 shows a correlation graph between CBR and percentages of cement. The 28 day water cured soaked CBR value increased with increased cement content from 10.5 % at 2 % cement to 25.8 % at 4 % cement to 49 % at 5 % cement.

**Table 6:** Variation of California Bearing Ratio values with cement content for Ekenkpon shale soil (28 days soaked)

	0 % Ordinary Portland cement	1 % Ordinary Portland cement	2 % of Ordinary Portland cement	3 % Ordinary Portland cement	4 % Ordinary Portland cement	5 % Ordinary Portland cement
CBR values after soaking (%)	15	–	10.5	20.5	25.8	49
Water Content before soaking (%)	16.5	–	18	18.1	18.2	16.1
Water content after soaking (%)	22.9	–	23.1	–	22.4	19.1
Maximum dry density (MDD) (kg/m^3^)	1700	1810	1710	1660	–	–
Optimum moisture content (OMC) (%)	16.6	19.8	18.12	18.11	–	–

## Shales from previous works

**Fig. 5:**
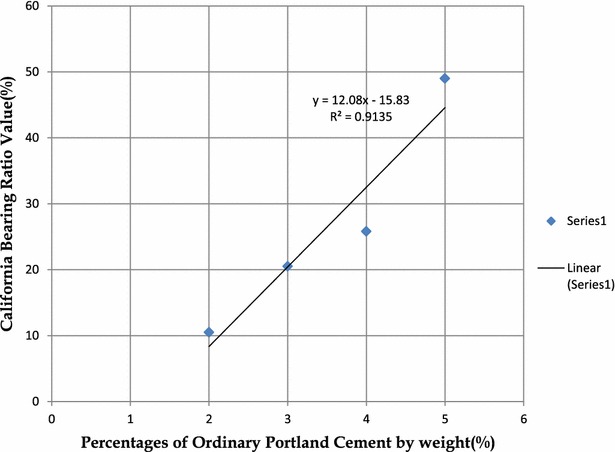
Variation of California Bearing Ratio values with various percentages of cement

Shale soil occurs in different parts of the world in various geologic settings. Their formation and environment of occurrence though varies, they possess unfavorable engineering characteristics and behavior which are similar with respect to civil engineering construction, in particular highway pavement construction. These include but are not limited to very high compressibility, low bearing strength, and expansiveness. Some soil indices from previous studies where the highway pavement were constructed on shale subgrade are presented in Table 7 with similar parameters for shales from this study also listed (Fig. 5).

**Table 7:** Soil indices and parameters for shale soil from other studies

Parameter	Nkporo shale	New Netim marl	Ekenkpon shale	Igumale^a^ shale	Abakaliki^b^ shale	Pulaski county, Kentucky, USA shale soil^c^
Fines (% passing sieve no 200)	80.1	68.4	81.4	90	90–92	29–46^f^
Plastic limit	30.0	33.0	38.4	27	17–23	18–23
Liquid limit	52.0	48.0	52.8	72	53–68	40-57
Plasticity index	22.0	15.0	14.4	45	36–45	22-34
Liquidity index^e^	−0.2	−1.2	−0.7	−0.04	0.14-0.11	0.02 to −0.08
Compression index Cc^d^	0.378	0.342	0.385	0.558	0.387–0.522	0.27–0.423
AASHTO classification	A-7-5(20)	A7-5(11)	A-7-5(15)	A-7-6	A-7-6	A-6(14) and A-7-6(25-27)
USCS classification	OH	MH	OH	CH	CH	CL and CH
Maximum dry density (Mg/m^3^)	1620.0	2020.0	1700.0	1510	1830–1890	N.L.
Optimum moisture content (%)	14.6	8.6	16.6	22	12.0–12.5	N.L.
CBR (%) (after 24 h soaking)	26.8	20.6	7.0	0.68	38–55	3
Free swell (%)	N.D	N.D	N.D.	50	48–52	N.L.
Specific gravity	N.D	N.D	N.D.	2.55	2.42–2.56	2.56 -2.79
Colour	Black	Yellowish	Dark grey	Grey	Bluish–Brownish	Red
Natural moisture content (%)	26.3	15.6	27.8	25	22-28	17.5–25.8
pH	2.27	6.97	4.28	6.83	N.L.	N.L.
Unconfined compressive strength (kN/m^2^)	784.19	674.53	350.57	400	N.L.	100.4

*N.D.* not determined, *N.L.* not listed by author

* In-situ value

^a^Manasseh and Olufemi (2008)

^b^Aghamelu and Okogbue (2011)

^c^Hopkins et al. (2006)

^d^Values from computed from Cc = 0.09 (LL − 10)

^e^Computed

^f^% finer than 0.002 mm (clay fraction)

The liquid limits of the shale in this study compared to the other two listed are not too widely different, however that of Igunmale shale at 72 % is far higher than all others listed. Closely associated with liquid limits is compressibility. This is based on compression index value Cc, for each of the shale and is computed using Terzaghi et al. (1996) relationship. Igunmale shale has the highest, with a value of 0.558, followed by the Abakaliki shale, and then shale from Pulaski County, Kentucky US, while Nkporo, Ekenkpon and New Netim marl all have smaller values. All classified into the ‘medium’ compressibility range based on the classification by McKinlay (1996).

The 24 h soaked CBR **v**alue is the parameter employed in assessing the bearing strength for the shales. None of the shales like the ones in this study meets the minimum requirement of 30 % except the Abakaliki shale. Igunmale shale has the lowest bearing strength with a soaked CBR value of 0.68 %, followed by the Kentucky soil with a value of 3 %. The unconfined compressive strength values follow a similar trend as the soaked CBR values, with a slight exception by the Igunmale shale.

In a geotechnical assessment of road failures in Abakaliki area of Southeastern Nigeria, Aghamelu and Okogbue (2011), attributed frequent failures of the roads studied due to the presence of expansive shale that forms the subgrade. With a plasticity index in the range of 36–45 % which puts the shale in the high expansive index based on the expansive potential classification presented in Table 8. The Igunmale shale as reported by Manasseh and Olufemi (2008), also has high expansive potential with a PI value of 45 %. They carried out X-ray diffraction analysis of the shale and showed it contain significant amount of Illite/Smectite, and pyrite among others. The Illite/Smectite clay minerals are the ones responsible for high potential for expansibility as they have high capacity to absorb a lot of water. In a study of soft soil in Kentucky in United States of America, Hopkins et al. (2006), reports on shale soil classified under AASHTO, as A-6(14) and A-7-6(25) with PI in the range of 22–34 % which is in the high expansive potential based also on the classification in Table 8. They report the presence of swelling of highway pavements constructed in the area. This is attributed to the expansive nature of subgrade soil. Compared to the shales in this study, only Nkporo shale can be said to be slightly expansive, the rest have low expansive potential.

**Table 8:** Classification of swelling potential

Seed et al. (1962) S^a^	Ola (1982) Plasticity index (PI) (%)	Ramana (1993) Liquid limit (LL) (%)	Terzaghi et al. (1996) Plasticity index	Expansive potential
0–1.567	0–15	<50	0–10	Low
1.567–5.45	15–25	50–70	10–20	Medium
5.45–12.39	25–35	71–90	20–35	High
>12.39	>35	>90	>35	Very high

^a^S = (2.116 × 10^−3^) (PI)^2.44^

In terms of natural color, they varied widely, from black to grey to yellowish and red. This coloration depends on the parent rock and the environment in which they are formed.

## Conclusions

Three dominant soils within a stretch of a highway alignment were characterized and stabilized. These three subgrade soils—Nkporo shale, Ekenkpon Shale, and New Netim Marl—do not meet basic requirements for subgrade materials for highway pavement. To improve them, Ordinary Portland Cement was investigated as a stabilizing agent for the three soils. These soils are all classified as A-7-5 soil with various values of the Group index. Based on strength and durability criteria (UCS value), 12, 7, and 3 % cement content by dry weight were respectively found to improve the soils properties such that they now meet requirements for subgrade and subbase construction materials. At optimum cement content in each case, LL was <40 %, and the PI was <10 % indicating non-swelling, stable soils. These results also indicate that for shale soils it is not sufficient to classify the soil (all classify as A-7-5) and hence make deductions from maybe previous study on the optimum cement content required for stabilization. The environment of deposition of the soil (alkaline or acidic) as indicated by the pH value for the soil also influences the optimum cement content. The optimum cement content required for stabilization increases with group index (GI) of the soil.

The optimum cement established by laboratory experiments can be achieved in the field using similar machines that have been used in previous cement stabilization operations. There are both ex situ and in situ methods. The ex situ methods involve removing subgrade soil to design depth to a plant site, drying it, mixing it with determined cement content through blending by machine, bringing the soil to the required moisture content, and laying the soil appropriately. The stationary mixing plant machines are an example of plant used to achieve this operation. The in situ method does the same operation but directly on location. Examples of machines used include windrow type, flat type and rotary mix-in place type, (O’Flaherty 2008; George 2001; Kowalski and Starry, 2007).

Compared with some other shales reported above, the shale in this study do not possess high expansive potential and are not as compressible. They however in their natural state have low bearing strength like these other shales. This constitutes a poor subgrade or potential problem for any pavement constructed on such.
